# Respiratory gas exchange as a new aid to monitor acidosis in endotoxemic rats: relationship to metabolic fuel substrates and thermometabolic responses

**DOI:** 10.14814/phy2.13100

**Published:** 2017-01-13

**Authors:** Alexandre A. Steiner, Elizabeth A. Flatow, Camila F. Brito, Monique T. Fonseca, Evilin N. Komegae

**Affiliations:** ^1^ Department of ImmunologyInstitute of Biomedical SciencesUniversity of São PauloSão PauloBrazil

**Keywords:** Acid buffering, glucose, hypothermia, inflammation, metabolism, respiratory exchange ratio, sepsis

## Abstract

This study introduces the respiratory exchange ratio (RER; the ratio of whole‐body CO_2_ production to O_2_ consumption) as an aid to monitor metabolic acidosis during the early phase of endotoxic shock in unanesthetized, freely moving rats. Two serotypes of lipopolysaccharide (lipopolysaccharide [LPS] O55:B5 and O127:B8) were tested at shock‐inducing doses (0.5–2 mg/kg). Phasic rises in RER were observed consistently across LPS serotypes and doses. The RER rise often exceeded the ceiling of the quotient for oxidative metabolism, and was mirrored by depletion of arterial bicarbonate and decreases in pH. It occurred independently of ventilatory adjustments. These data indicate that the rise in RER results from a nonmetabolic CO_2_ load produced via an acid‐induced equilibrium shift in the bicarbonate buffer. Having validated this new experimental aid, we asked whether acidosis was interconnected with the metabolic and thermal responses that accompany endotoxic shock in unanesthetized rats. Contrary to this hypothesis, however, acidosis persisted regardless of whether the ambient temperature favored or prevented downregulation of mitochondrial oxidation and regulated hypothermia. We then asked whether the substrate that fuels aerobic metabolism could be a relevant factor in LPS‐induced acidosis. Food deprivation was employed to divert metabolism away from glucose oxidation and toward fatty acid oxidation. Interestingly, this intervention attenuated the RER response to LPS by 58%, without suppressing other key aspects of systemic inflammation. We conclude that acid production in unanesthetized rats with endotoxic shock results from a phasic activation of glycolysis, which occurs independently of physiological changes in mitochondrial oxidation and body temperature.

## Introduction

Although it is recognized that acidosis occurs in septic patients and may even predict clinical outcome (Gunnerson et al. 2006; Park et al. 2006; Lee et al. 2008), the pathogenesis of acidosis in inflammatory states is still poorly understood. In clinical sepsis, acidosis has many overlapping causes, which include not only the metabolic release of H^+^ but also the administration of electrolyte solutions as well as respiratory and renal derangements (Kellum 2004; Gunnerson et al. 2006; Noritomi et al. 2009; Mallat et al. 2012). Experimentally though, metabolic H^+^ production can be studied in the absence of the other confounding factors during the early phase of hypodynamic endotoxic shock – lipopolysaccharide (LPS)‐induced shock without fluid resuscitation. It is generally thought that acidosis in early endotoxic shock results from the anaerobic production of lactic acid by hypoperfused (hypoxic) tissues, but this idea has been challenged by three lines of evidence. First, improvement of hemodynamics and tissue oxygenation in the endotoxic shock model often fails to attenuate mucosal H^+^ production and acidosis (Schaefer et al. 1995; VanderMeer et al. 1995; Oldner et al. 1999; Aksu et al. 2012). Second, there are reports of LPS‐induced acidosis being dissociated from hyperlactatemia (Parratt and Sturgess 1974; Kellum et al. 1997). Third, despite the widespread acceptance of the lactic acidosis theory, there is no biochemical evidence indicating that lactate can actually contribute to metabolic acidosis. On the contrary, there is evidence that lactate is produced as a salt, and that its production via lactate dehydrogenase consumes H^+^ (Robergs et al. 2004).

These lines of evidence raise the question as to whether heightened H^+^ production in endotoxic shock could result from metabolic alterations unrelated to anaerobic metabolism. From this perspective, it is relevant to point out that whole‐body oxygen consumption ($\dot{V}$O_2_) is known to fall in conjunction with core temperature (T_c_) early in the course of endotoxic shock in unanesthetized rats (Derijk et al. 1994; Romanovsky et al. 1996; Garrett‐Cox et al. 2003) as well as in chicks (Dantonio et al. 2016). Recently, Corrigan et al. (2014) have shown that, at least in rats, this hypometabolic, hypothermic response occurs when neither oxygen delivery nor mitochondrial function is impaired to the point of compromising aerobic metabolism. The same study has revealed that the fall in $\dot{V}$O_2_ occurs preemptively and in synchrony with the fall in oxygen delivery, thus preventing tissue hypoxia. Here, we hypothesized that this thermometabolic response could have an impact on H^+^ production during endotoxic shock. Undeniably, changes in mitochondrial respiration and temperature have the potential to interact multilaterally with a diversity of metabolic processes, including those responsible for the net release of H^+^, that is, the steps of glycolysis upstream of lactate production and the hydrolysis of ATP (Robergs et al. 2004). However, the relevance and the nature of such interactions vary substantially depending on cell phenotypes and experimental paradigms (Scaion and Sebert 2008; Cabrera et al. 2012; Cruz et al. 2012; Martin et al. 2012; Molenaar et al. 2013; Liemburg‐Apers et al. 2015; Offer and Ranatunga 2015; Satrustegui and Bak 2015).

Another factor that can impact H^+^ production independently of anaerobic metabolism is the chemical nature of the metabolic fuel substrate. Unlike glucose oxidation, fatty acid oxidation does not result in a net release of H^+^ (Robergs et al. 2004). Therefore, situations that favor the use of fatty acids instead of glucose (like food deprivation) would be expected to mitigate metabolic acidosis if glycolysis happens to be the main source of H^+^ in endotoxic shock, but not if this source happens to be ATP hydrolysis. This postulate provides the basis for employing food deprivation as a tool to identify the metabolic source of H^+^ in endotoxic shock. It should be considered, however, that food deprivation should be kept as short as possible, as extended periods (usually >2 days) without feeding are known to result in the production of acidic ketone bodies (Berg et al. 2002; Kamel and Halperin 2015).

In light of these arguments, this study had two goals: (1) to evaluate whether there is a causal relationship between thermometabolic responses and acidosis in endotoxic shock; and (2) to investigate whether the utilization of glucose versus fatty acids has any impact on the acidosis of endotoxic shock. To achieve these goals, we developed a method to frequently monitor H^+^ production in unanesthetized, freely moving rats during endotoxic shock. The method is based on the respiratory exchange ratio (RER), the ratio of whole‐body CO_2_ production ($\dot{V}$CO_2_) to $\dot{V}$O_2_. In steady‐state conditions, RER typically reflects the respiratory quotient (Peronnet and Massicotte 1991), that is, the stoichiometry of CO_2_ produced to O_2_ consumed by the metabolic oxidation of glucose (quotient of 1.0) versus fatty acids (quotient of 0.7). However, acute rises in RER more commonly reflect CO_2_ originated from equilibrium shifts in the bicarbonate buffer, like the shift caused by H^+^ buffering in exercise (Naimark et al. 1964; Beaver et al. 1986). Isolated increases in alveolar ventilation may also produce a rise in RER, but in that case, excess removal of CO_2_ from the blood results in alkalosis (Jack et al. 2004). Hence, in order to use RER as a correlate of acidosis in endotoxic shock, we first had to validate changes in this variable against changes in pH and related parameters.

## Methods

### Animals

The study was conducted in 70 male Wistar rats obtained from the pathogen‐free breeding facility of the Institute of Biomedical Sciences (University of São Paulo, São Paulo, Brazil). The rats were grouped 2–3 per cage prior to surgery and singly after surgery. The animal room was under a 12:12 h light–dark cycle (lights on at 7:00 am), and its ambient temperature was maintained between 24 and 27°C. Filtered water was available ad libitum. Standard chow was also freely available, except for a 24‐h food deprivation period in one of the experimental groups (see Experimental interventions). The rats weighed an average of 290 g on the day of the experiments. Immediately after the end of an experiment, each rat was killed with an intravenous (IV) overdose of sodium thiopental (100 mg/kg). All protocols were approved by the Animal Care and Use Committee at the Institute of Biomedical Sciences of the University of São Paulo (São Paulo, Brazil).

### Surgical preparation

In preparation for an experiment, a rat was chronically implanted, as necessary, with an IV catheter (later used for LPS administration), an intraarterial (IA) catheter (later used for blood collection), and an abdominal telemetry transmitter (later used for measurements of T_c_ and arterial pressure). Surgery was performed under anesthesia with isoflurane (1.5–2.5%) and antibiotic protection with enrofloxacin (5 mg/kg, subcutaneously). The rats were maintained on a heated (37–39°C) operating board for the duration of surgery.

The IV catheter was inserted into the left jugular vein, and advanced so that its tip reached the right atrium. The IA catheter was inserted into the left carotid artery, and advanced so that its tip reached the descending aorta. The catheters consisted of 3‐Fr polyurethane tubing. They were secured in place by occlusive ligatures, exteriorized at the nape, and locked with heparinized glycerol (500 U/mL).

The telemetry transmitter (Data Sciences International, St. Paul, MN) was introduced in the peritoneal cavity via a midline laparotomy. A TA‐F40 transmitter was employed when only T_c_ was to be measured, whereas a C50‐PT transmitter was employed when both T_c_ and arterial pressure were to be measured. The TA‐F40 transmitter was left loose in the peritoneal cavity. The C50‐PT transmitter consisted of a capsule and a gel‐filled catheter, of which the latter was implanted in the abdominal aorta. While the catheter was inserted and secured in place with surgical glue, the aorta was occluded caudally to the renal arteries for 90 seconds or less. No permanent ligatures were used in this procedure.

All incision sites were closed in layers. Ketoprofen (5 mg/kg, subcutaneously) was administered for pain management at the end of surgery and on the next day. The rats were allowed to recover for 7–10 days before an experiment. During the recovery period, the catheters were flushed with saline and relocked with heparinized glycerol on the first day post surgery and every third day thereafter.

### Experimental interventions

Rats designated for food deprivation had their feeder emptied at 8:00 am on the day prior to the experiment, and were placed on an elevated metal grid floor to preclude coprophagy. Control rats had access to food ad libitum. After 24 h under either of these conditions, each rat was transferred to an experimental cage containing a thin layer of bedding (<1 cm). Chow continued to be denied or provided for the duration of the experiment, which typically ended around 6:00 pm. Water was freely available at all times.

The experimental cages were housed inside an environmental chamber (NQ1 model; Environmental Growth Chambers, Chagrin Falls, OH), which provided precise control of the ambient temperature (T_a_) and air humidity. At relative humidity of 40–60%, the lower limit of the thermoneutral zone for healthy rats in this experimental setup was found to be 25°C, as determined based on the influence of T_a_ on resting $\dot{V}$O_2_; for details on this method, see Gordon (1990). The experiments were conducted at two T_a_s: one (22°C) that is mildly below and another (29°C) that is mildly above the lower limit of the thermoneutral zone. The lower T_a_ was chosen as a condition in which nonshivering thermogenesis is activated at rest and can be inhibited during endotoxic shock, thus resulting in hypometabolism and hypothermia. Such cool environments are preferred by rats during the early stage of endotoxic shock (Romanovsky et al. 1996; Almeida et al. 2006a,b). On the other hand, the higher T_a_ was used to keep thermogenesis at a minimum during the whole experiment, thus leaving no room for thermometabolic inhibition and development of hypothermia. In such warm environments, fever brought about by cutaneous vasoconstriction develops even in the most severe cases of endotoxic shock (Szekely and Szelenyi 1979; Romanovsky et al. 1997, 1998; Carroll et al. 2001; Boisse et al. 2004; Krall et al. 2010; Liu et al. 2012; Corrigan et al. 2014).

Systemic inflammation was induced by two LPS serotypes (O55:B5 and O127:B8) at nonlethal doses (0.5–2 mg/kg) known to induce endotoxic shock in rats (Lang et al. 1985; Romanovsky et al. 1996; Miura et al. 2000; Yilmaz et al. 2008a,b; Steiner et al. 2009; Corrigan et al. 2014). The effectiveness of these doses was confirmed by their ability to induce hypotension (see, e.g., Fig. 8). Both serotypes were obtained from Sigma‐Aldrich (St. Louis, MO), suspended in sterile, pyrogen‐free saline at a concentration of 5 mg/ml, and stored at 4°C for no longer than 3 months. On the day of the experiment, the stock was warmed up to room temperature, diluted to a concentration of 0.5, 1.0, or 2.0 mg/mL, and injected in bolus at 1 mL/kg between noon and 1 pm. Control rats were injected with saline (1 mL/kg) in the same time window. All injections were made from outside the environmental chamber via PE50 extensions of the IV catheters, without disturbing the animals. Each extension exited the rat's experimental cage via a 2.5‐cm hole on its acrylic lid, was passed by a swivel system (Instech Laboratories, Plymouth Meeting, PA), and its distal end was brought to the exterior of the environmental chamber via a wall port. A coil spring connected to an infusion harness worn by the rat provided torque to move the swivel, while also protecting the catheter extension from bites and scratches.

### Measurements

$\dot{V}$O_2_ and $\dot{V}$CO_2_ were measured by flow‐through respirometry using equipment from Sable Systems (Las Vegas, NV). The experimental cage was made of acrylic and had a volume of 5 liters. A 2.5‐cm hole in its lid served as the air input; hose connectors on its sides served as the air output. Air was pulled from the side connectors at a rate of ~1700 mL/min (STP) with the help of an adjustable pump coupled to a mass‐flow controller (SS4 unit). The air was dehumidified by a Drierite column before entering the pump. The output air of a rat‐bearing cage (expired air) and that of an empty cage (inspired air) were subsampled sequentially at 5‐min intervals and analyzed for the fraction of O_2_ and CO_2_ with the help of a channel multiplexer and a FOXBOX gas analyzer. Due to the tubing dead space, it took 10 sec for the air sample to reach the gas analyzer, but this time lag was corrected during data processing. The analog outputs of the mass‐flow controller (air flow data) and of the gas analyzer (gas fraction data) were converted to digital by the UI‐2 data acquisition interface, and acquired by a computer using the Expedata software. Under these experimental conditions, the fraction of O_2_ in the rat‐bearing cage never fell below 19.5%, whereas the fraction of CO_2_ never went above 0.4%.

T_c_ and arterial pressure were measured by telemetry (Data Sciences International). The radio waves emitted by the preimplanted abdominal transmitters were captured by a PhysiolTel RPC‐1 receiver located under the experimental cage. The signal of each transmitter was processed by a Data Exchange Matrix and forwarded to a computer, where data acquisition was handled by the Dataquest ART software. Data were acquired in continuous mode, and later processed in Ponemah.

The pH and the partial pressure of CO_2_ in arterial blood **(** *P*_a_CO_2_) were measured using the iSTAT‐1 portable gas analyzer equipped with EG7^+^ cartridges (Abbott Point of Care, Abbott Park, IL). These cartridges also provided readings of the plasma concentrations of sodium ([Na^+^]_pl_) and potassium ([K^+^]_pl_). The analysis required less than 100 *μ*L of blood, which was dripped directly from the IA catheter extension into the collection chamber of the cartridge. Immediately after this procedure, blood that remained in the catheter extension was flushed back into the animal with saline. No more than four serial samples were collected during an experiment. The IA catheter extension ran in parallel to the IV line employed for LPS administration (see Experimental interventions), each passing through an independent channel of a two‐channel swivel (Instech).

### Derived parameters

$\dot{V}$CO_2_ and $\dot{V}$O_2_ (in mL/kg/min) were calculated from the gas fractions in inspired air (F_i_CO_2_ or F_i_O_2_) and expired air (F_e_CO_2_ or F_e_O_2_), taking into consideration the air flow ($\dot{Q}$, in mL/min at STP) and the animal's body mass (BM, in kg). Equations 1 and 2 were used to account for the unequal volumes of CO_2_ breathed out and O_2_ breathed in (Lighton 2008). $\dot{V}$CO_2_ was divided by $\dot{V}$O_2_ to obtain RER.

(1) $$\dot{V}CO_{2}=\dot{Q}\times \left[\left(F_{e}CO_{2}-F_{i}CO_{2}\right)+F_{i}CO_{2}\times \left(F_{i}O_{2}-F_{e}O_{2}\right)\right]/\left(1+F_{i}CO_{2}\right)/\text{BM}$$

(2) $$\dot{V}O_{2}=\dot{Q}\times \left[\left(F_{i}O_{2}-F_{e}O_{2}\right)-F_{i}O_{2}\times \left(F_{e}CO_{2}-F_{i}CO_{2}\right)\right]/\left(1-F_{i}O_{2}\right)/\text{BM}$$

The concentration of bicarbonate in arterial blood ([HCO_3_^−^]_a_, in mM) was calculated from pH and P_a_CO_2_ (in mmHg) by rearranging the terms of the Henderson–Hasselbalch equation (Equation 3). As an estimate of the overall amount of titratable base in the extracellular fluid, standard base excess (SBE, in mM) was calculated from [HCO_3_^−^]_a_ and pH using the Van Slyke equation (Equation 4). These equations are in conformity with the guidelines of the Clinical and Laboratory Standards Institute (CLSI 2001). Lastly, alveolar ventilation ($\dot{V}$_A_, in mL/kg/min) was derived from $\dot{V}$CO_2_ (in mL/kg/min) and P_a_CO_2_ (in mmHg) according to Equation 5 (Martin 1987).

(3) $$\text{log}{\left[\text{HC}{O_{3}}^{-}\right]}_{a}=\text{pH}+\text{log}P_{a}CO_{2}-7.608$$

(4) $$\text{SBE}={\left[\text{HC}{O_{3}}^{-}\right]}_{a}-24.8+16.2\times \left(\text{pH}-7.4\right)$$

(5) $${\dot{V}}_{A}=\dot{V}CO_{2}\times 863/P_{a}CO_{2}$$

### Statistical analyses

Statistical comparisons were performed using Statistica Advanced 8.0 (StatSoft, Tulsa, OK), with the level of significance set at *P *<* *0.05. Changes in RER, $\dot{V}$O_2_, T_c_, and arterial pressure were evaluated across the different interventions and time points by a mixed‐model ANOVA, with time set as a within‐subjects factor (repeated measures) and interventions as a between‐subjects factor. One‐way ANOVA for repeated measures was employed to evaluate how pH or a related parameter differed before and after LPS administration. The related parameters evaluated were as follows: P_a_CO_2_, [HCO_3_^−^]_a_, SBE, $\dot{V}$CO_2_, $\dot{V}$_A_, [K^+^]_pl_, and [Na^+^]_pl_. The different types of ANOVA were followed, as necessary, by the Fisher least significant difference post hoc test. Student's *t*‐test for a single sample was used to determine 95% confidence intervals for the amplitude of the RER responses. A *t*‐test for two independent samples was employed to assess the effects of food deprivation (compared to unrestricted feeding) on the basal levels of RER, averaged over a 30‐min interval.

## Results

### A rise in RER is a correlate of acidosis during early endotoxic shock in unanesthetized rats

**Figure 1:**
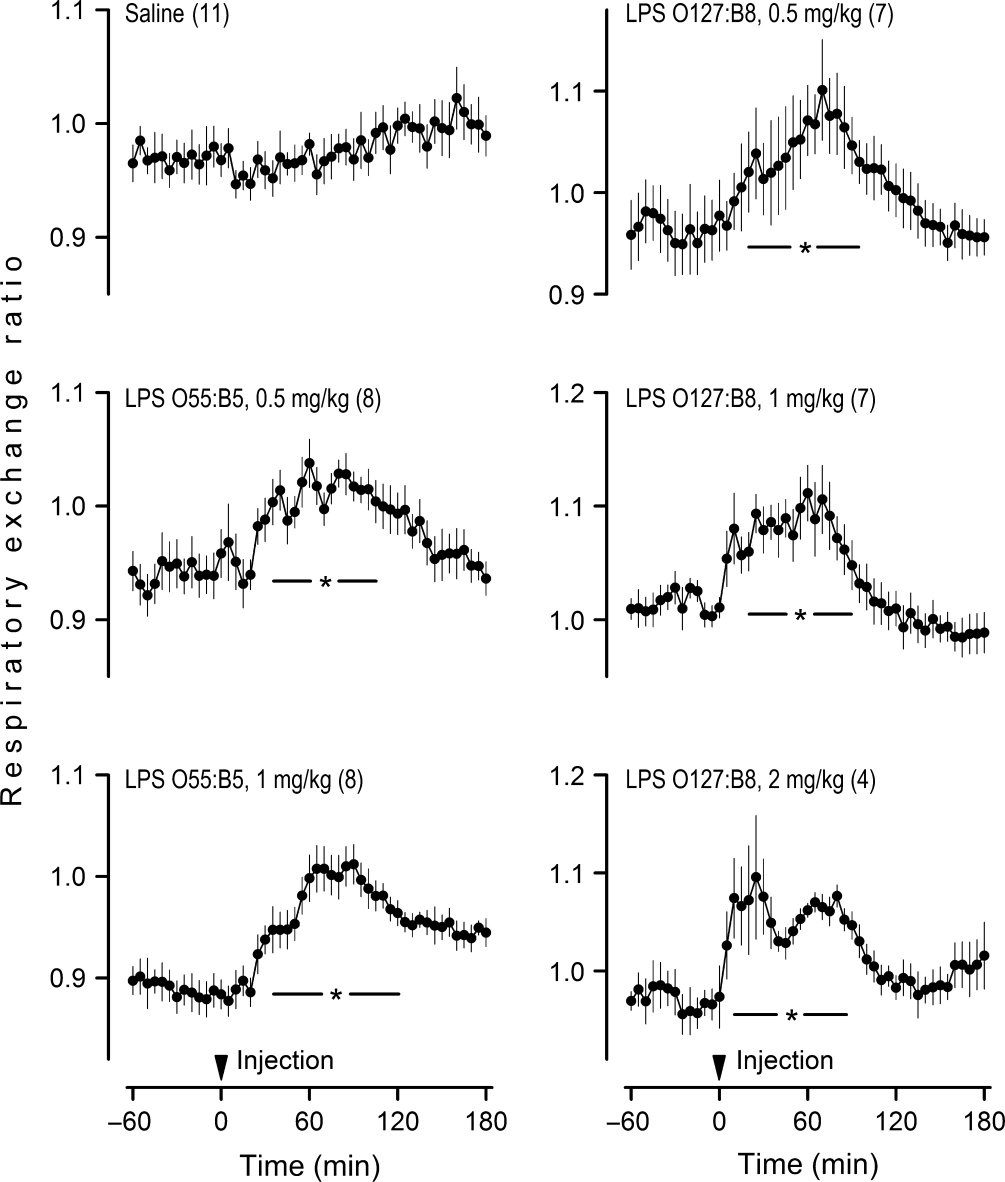
Unanesthetized rats respond to shock‐inducing doses of lipopolysaccharide (LPS) with phasic rises in the respiratory exchange ratio. Rats in this experiment were exposed to an ambient temperature of 22°C. LPS or its vehicle (saline) was administered via an IV catheter extension, without disturbing the rat. LPS serotypes and doses are indicated. The arrowhead marks the injection time. Data are shown as means ± standard errors. The number of animals in each group is shown in parentheses. The asterisk‐containing bar denotes the time window in which an LPS‐injected group was statistically different (*P *<* *0.05) from the saline‐injected (control) group.

This initial series of experiments was conducted in free‐feeding rats at a T_a_ of 22°C. Two LPS serotypes (O55:B5 and O127:B8) were tested at shock‐inducing doses (0.5, 1, and 2 mg/kg). None of the doses were lethal. All serotype‐dose combinations induced statistically significant rises in RER, as compared to the relatively constant RER of the saline‐injected controls (Fig. 1). The RER response to LPS was transient, not lasting longer than 125 min in any of the experimental groups. The time for the onset of the response was ~20 min when the LPS dose was 0.5 or 1 mg/kg, but an early onset at ~5 min was observed when the dose was 2 mg/kg. The response to the highest dose was also unique in having a biphasic pattern. The amplitude of the RER rise was remarkably consistent, not differing across LPS serotypes and doses; it averaged 0.12 across all groups (95% confidence interval from 0.09 to 0.14). During the course of a response, it was not uncommon for RER to reach values higher than 1.00.

**Figure 2:**
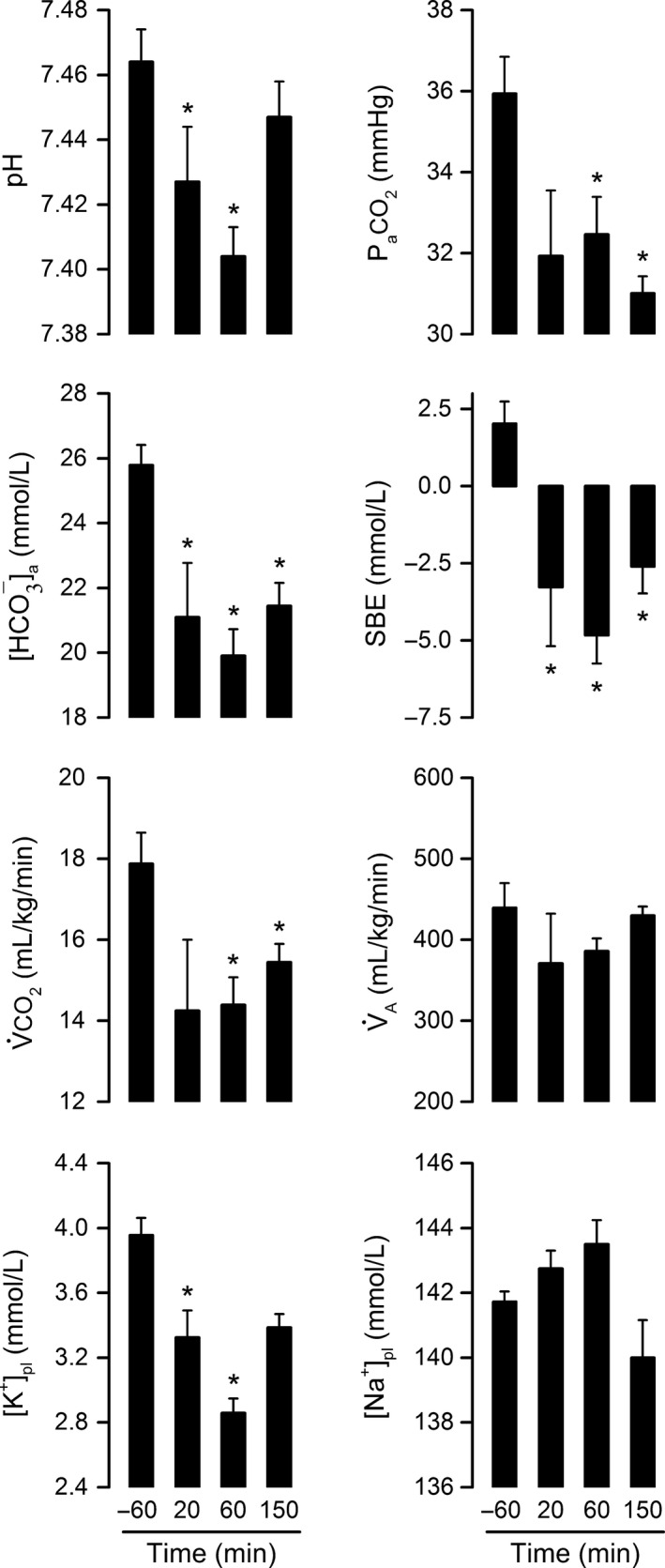
Metabolic acidosis matches the course of the lipopolysaccharide (LPS)‐induced rise in respiratory exchange ratio. Rats in this experiment were exposed to an ambient temperature of 22°C. LPS O55:B5 was administered at the dose of 0.5 mg/kg via an IV catheter extension. The pH of the arterial blood and related parameters were assessed at four crucial time points of the respiratory exchange ratio response: −60 min (baseline), 20 min (response onset), 60 min (response peak), and 150 min (post response). The related parameters assessed were as follows: P_a_ CO_2_, arterial partial pressure of CO_2_; [HCO_3_^−^]_a_, bicarbonate concentration in arterial blood; SBE, standard base excess; V̇CO_2_, whole‐body CO_2_ production; $\dot{V}$_A_, alveolar ventilation; [K^+^]_pl_, plasma concentration of potassium; and [Na^+^]_pl_, plasma concentration of sodium. Data are shown as means ± standard errors. Sample sizes were 11–12 at −60 min, 3–4 at 20 min, 10–12 at 60 min, and 7 at 150 min. Each asterisk denotes a statistical difference (*P *<* *0.05) from the baseline value (−60 min).

To investigate whether acid–base disturbances accounted for the LPS‐induced rise in RER, arterial pH and related parameters were determined in rats injected with the 0.5 mg/kg dose of LPS O55:B5 at four stages of the RER response: −60 min (baseline), 20 min (response onset), 60 min (response peak), and 150 min (post response). The results clearly show that the course of acidosis mirrors the course of the RER response. More specifically, pH was progressively reduced from 20 to 60 min post LPS, after which it returned toward baseline levels (Fig. 2). This transient acidosis was not of respiratory origin, since P_a_CO_2_ was not increased, but rather reduced. The reduction in P_a_CO_2_ occurred in the absence of changes in V_A_; it presumably reflected a diminished $\dot{V}$CO_2_. The fact that pH fell in conjunction with [HCO_3_^−^]_a_ and SBE characterizes the disturbance as metabolic acidosis. The magnitude of the acid–base shift was mild: at the peak of the response (60 min), pH was reduced by 0.060 and [HCO_3_^−^]_a_ was reduced by 5.9 mmol/L. Hence, RER appears to be a very sensitive index of H^+^ production in endotoxemia. Regarding the recovery phase of the response (60–150 min), it is interesting that the fall in [HCO_3_^−^]_a_ was not fully reversed, even though the change in pH was. Although this may seem odd at first glance, it actually makes sense if we consider pH as the regulated variable. More specifically, given that the hypometabolic response held P_a_CO_2_ down as the rats recovered from acidosis, [HCO_3_^−^]_a_ had to be maintained at a lower level in order to avoid overcompensation and alkalosis.

Figure 2 also shows that LPS‐induced acidosis was associated with a decrease in [K^+^]_pl_ (hypokalemia), while osmoregulatory mechanisms maintained [Na^+^]_pl_ unaltered. Development of hypokalemia in this case is consistent with the known increase in the renal excretion of K^+^ that accompanies hypotensive, hypodynamic states (Jacobson and Seldin 1983), which correspond to the state of animals in the experimental model of endotoxic shock without fluid supplementation (Corrigan et al. 2014). And since K^+^ loss is typically coupled with H^+^ loss in these cases (Jacobson and Seldin 1983), it seems unlikely that the kidneys contributed to the genesis of acidosis in this study.

### Metabolic acidosis in endotoxic shock occurs independently of thermometabolic responses

**Figure 3:**
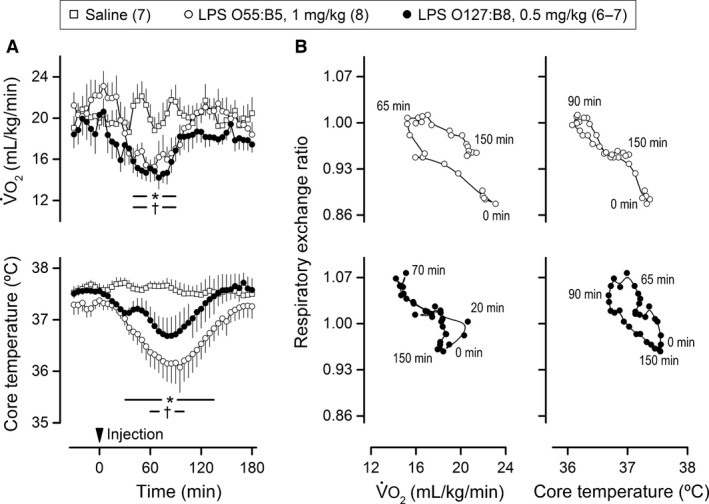
The lipopolysaccharide (LPS)‐induced rises in respiratory exchange ratio are not consistently synchronized with the hypometabolic, hypothermic response. The experiment was conducted at an ambient temperature of 22°C. LPS or its vehicle (saline) was administered via extensions of IV catheters; LPS serotypes and doses are indicated. In (A), means** **± standard errors for whole‐body oxygen consumption ($\dot{V}$O_2_) and core temperature are plotted as a function of time. The arrowhead marks the injection time. The numbers of animals in the experimental groups are shown in parentheses. The asterisk‐containing and the cross‐containing bars denote statistical difference (*P *<* *0.05) in the groups injected with LPS O55:B5 and LPS O127:B8, respectively, as compared to the saline‐injected controls. In (B), phase‐plane plots are shown for the temporal relationships of the respiratory exchange ratio with either $\dot{V}$O_2_ or core temperature. The first point in each graph is from the time of the LPS injection (0 min), whereas the last point is from the time corresponding to the end of the responses of interest (150 min).

Regardless of serotype or dose, LPS induced transient, self‐limiting falls in $\dot{V}$O_2_ and T_c_ in rats exposed to a T_a_ of 22°C (Fig. 3A). The change in $\dot{V}$O_2_ preceded the change in T_c_ during both the onset and recovery phases of the responses. To probe for temporal relationships between acidosis and thermometabolic responses, we created phase‐plane plots of RER as a function of $\dot{V}$O_2_ or T_c_. Parameters with demonstrated cause–effect relationships form loops in this type of plot. A clockwise loop appeared to exist in the RER x $\dot{V}$O_2_ plot for the effects of LPS O55:B5, but this relationship was not confirmed for LPS O127:B8 (Fig. 3B). By the same token, a counterclockwise loop was formed in the RER x T_c_ plot for the effects of LPS O127:B8, but the result was not reproduced for the other LPS serotype.

**Figure 4:**
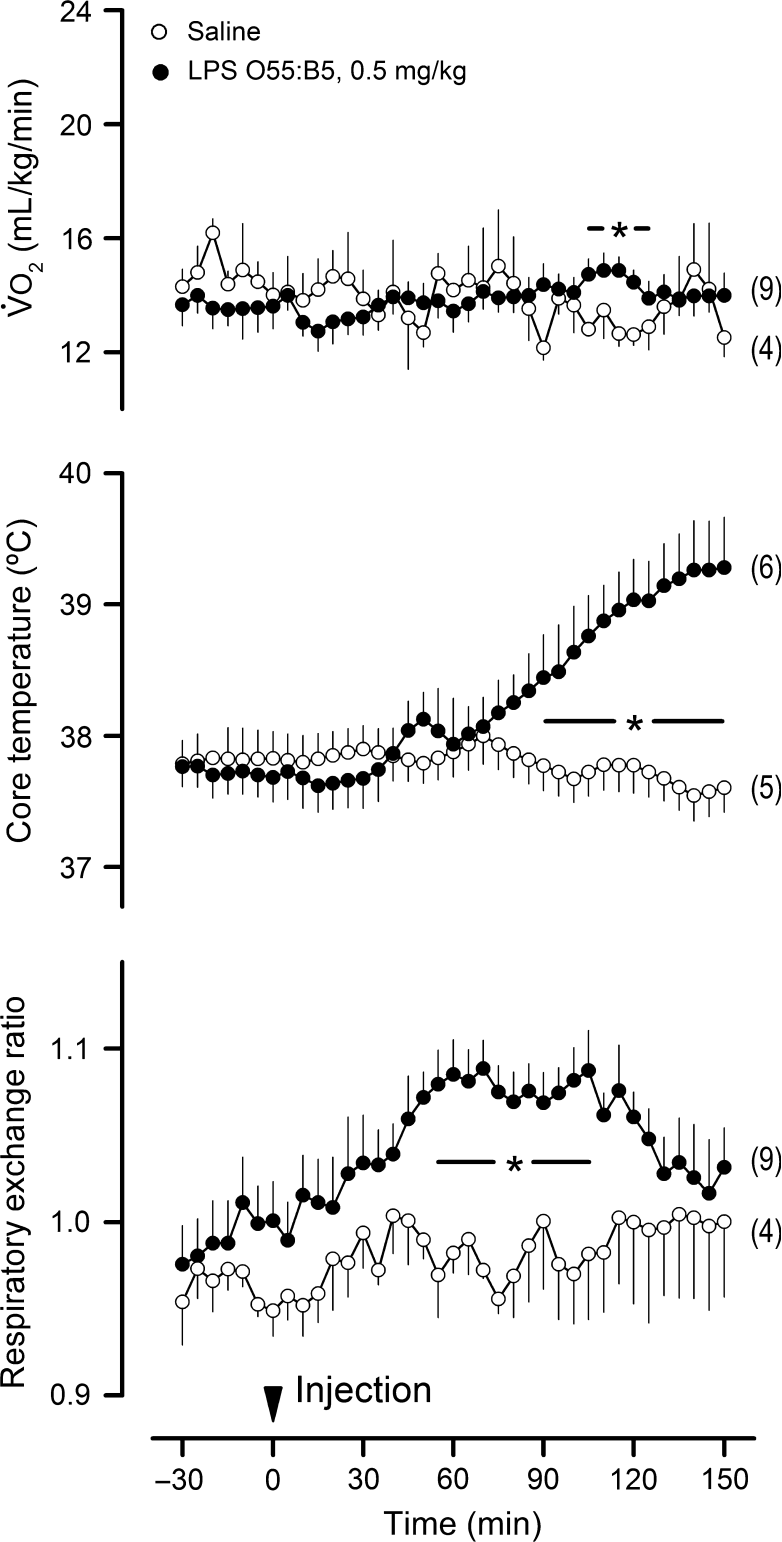
The lipopolysaccharide (LPS)‐induced rise in respiratory exchange ratio persists in the absence of the hypometabolic, hypothermic response at an ambient temperature of 29°C. As in the other experiments, IV catheter extensions were used for the administration of LPS (serotype and dose indicated) or its vehicle (saline). The arrowhead marks the injection time. Data are shown as means ± standard errors. The number of animals in each group is shown in parentheses. The asterisk‐containing bar denotes the time window in which the LPS‐injected group was statistically different (*P *<* *0.05) from the saline‐injected (control) group.

**Figure 5:**
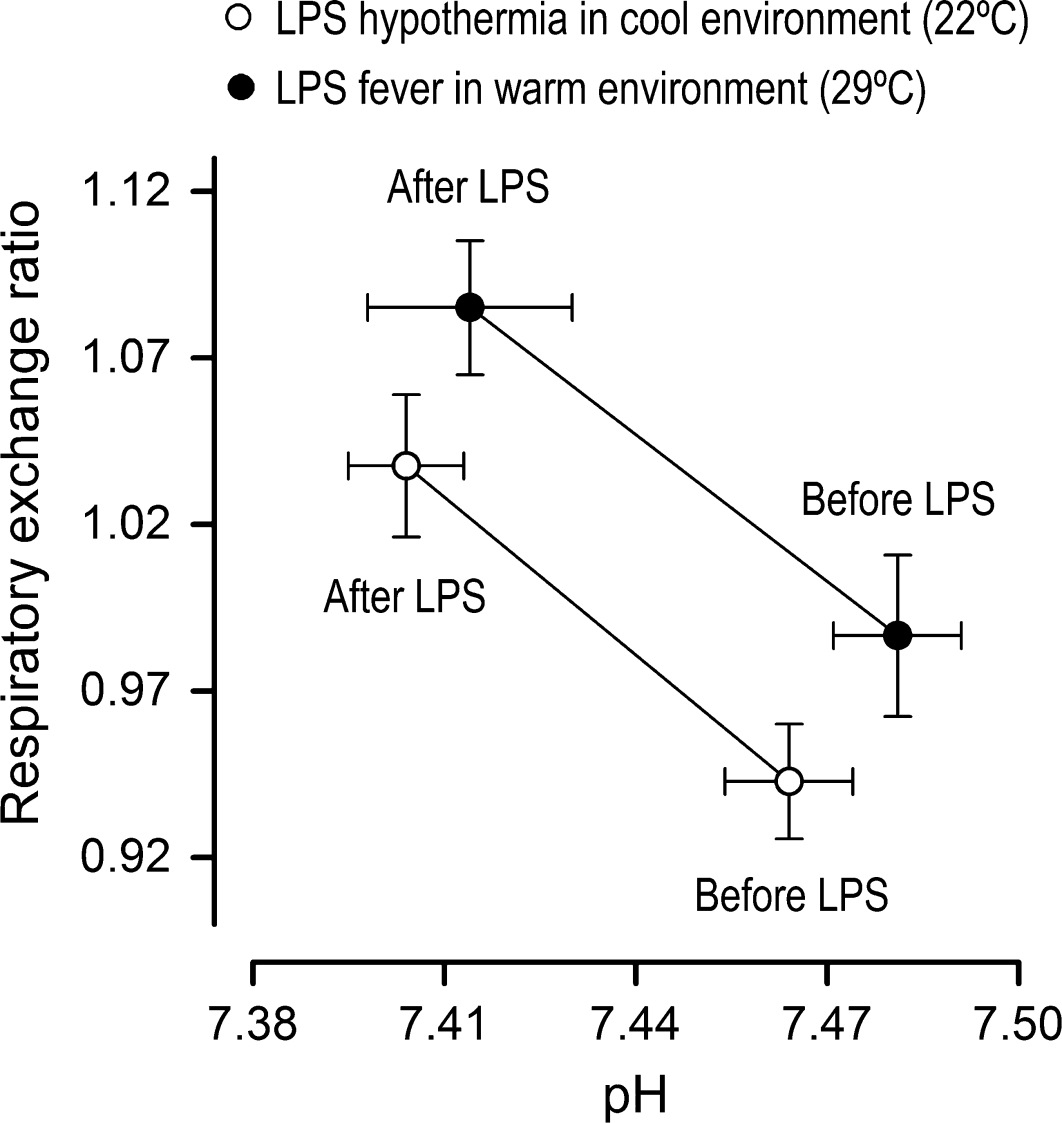
Changes in core temperature do not affect the proportionality of the lipopolysaccharide (LPS)‐induced changes in respiratory exchange ratio and pH. This analysis was made for the responses to LPS O55:B5 at the shock‐inducing dose of 0.5 mg/kg. Two groups of rats with opposite core temperature responses were compared: hypothermia at an ambient temperature of 22°C versus fever at an ambient temperature of 29°C. Means ± standard errors are shown for the data obtained at 60 min before LPS and 60 min after LPS.

**Figure 6:**
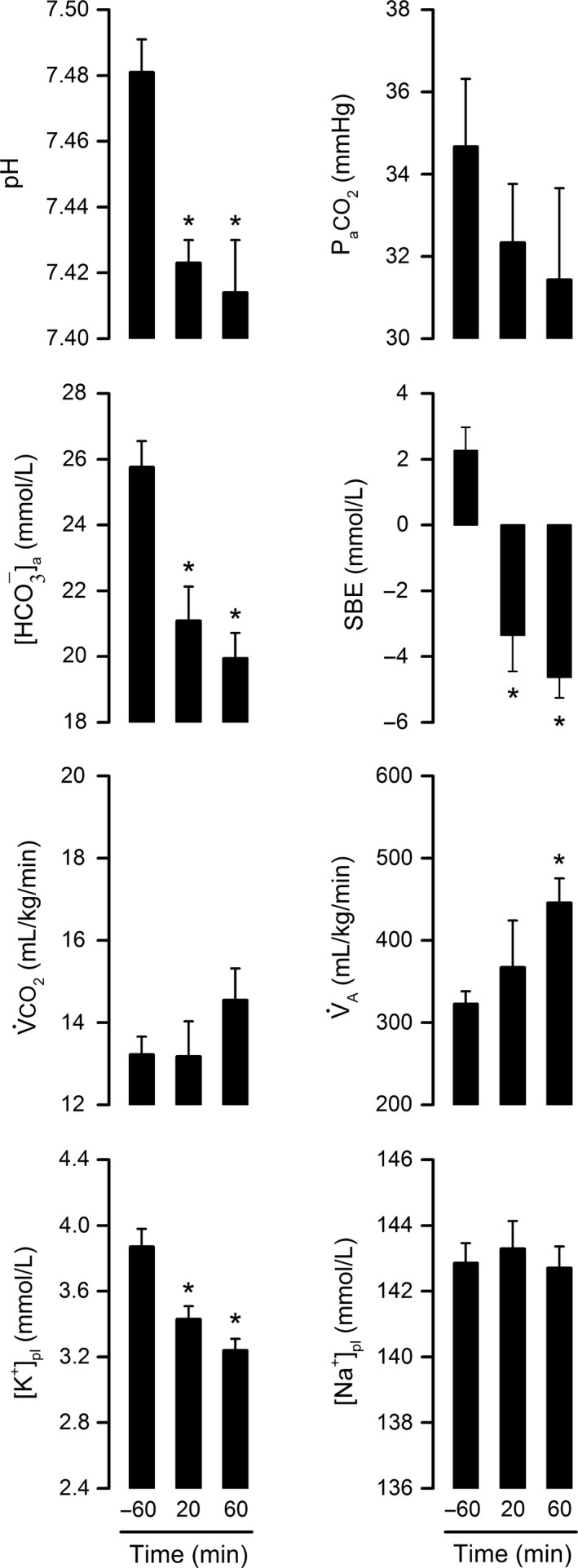
Characteristics of lipopolysaccharide (LPS)‐induced metabolic acidosis in the warm environment (ambient temperature of 29°C). LPS O55:B5 was administered at the dose of 0.5 mg/kg via an IV catheter extension. Arterial blood was sampled at three crucial time points of the respiratory exchange ratio response: −60 min (baseline), 20 min (response onset), and 60 min (response peak). In addition to pH, the following parameters were assessed: P_a_ CO_2_, arterial partial pressure of CO_2_; [HCO_3_^−^]_a_, bicarbonate concentration in arterial blood; SBE, standard base excess; V̇CO_2_, whole‐body CO_2_ production; $\dot{V}$_A_, alveolar ventilation; [K^+^]_pl_, plasma concentration of potassium; and [Na^+^]_pl_, plasma concentration of sodium. Data are shown as means ± standard errors. Sample size was 5–7 for all time points. Each asterisk denotes a statistical difference (*P *<* *0.05) from the baseline value (−60 min).

Our next step was to test in a direct experiment whether prevention of the hypometabolic, hypothermic response to LPS would impact the RER response. This was achieved by exposure to a warm environment (T_a_ of 29°C). This strategy has been employed in several previous studies (Steiner et al. 2004, 2009; Krall et al. 2010; Liu et al. 2012; Corrigan et al. 2014), and is based on the premise that shock‐inducing doses of LPS cause hypothermia by lowering the T_c_ threshold for activation of cold‐defense effectors, without reducing the T_c_ threshold for activation of heat‐defense effectors (Romanovsky et al. 1996). Accordingly, rats exposed to 29°C presented no reduction in $\dot{V}$O_2_ when injected with LPS O55:B5 at the dose of 0.5 mg/kg (Fig. 4). In the absence of hypometabolism, the prevailing T_c_ response changed from hypothermia to fever, and $\dot{V}$O_2_ was even increased for a brief period during the febrile response. Remarkably, in sharp contrast to $\dot{V}$O_2_ and T_c_ responses, the RER response to LPS remained unaltered at the T_a_ of 29°C (Fig. 4), in comparison with the RER response to the same LPS serotype and dose at the T_a_ of 22°C (Fig. 1). The proportionality of the inverse relationship between RER and pH in early endotoxic shock was also unaltered by the modification of T_a_ (Fig. 5). Other T_a_‐independent factors were [HCO_3_^−^]_a_, SBE, [K^+^]_a_, and [Na^+^]_a_ (Fig. 6).

In contrast, the interplay among P_a_CO_2_, $\dot{V}$CO_2_ and $\dot{V}$_A_ was dependent on T_a_ (Fig. 6). In the warm environment, $\dot{V}$CO_2_ was no longer the main determinant of P_a_CO_2_, and an elevation in V_A_ was associated with a trend toward a reduction in P_a_CO_2_ (Fig. 6). The elevation in V_A_ might reflect the tachypnea that often accompanies endotoxemia, a response that could have been favored by the absence of hypothermia in the warm environment. The mechanisms behind this respiratory response are unknown, but it has been shown in anesthetized cats with T_c_ artificially maintained at 38°C that LPS‐induced tachypnea depends on signals triggered in the carotid bodies via an unconventional, hypoxia‐independent mechanism (Fernandez et al. 2008). An intrinsic immunosensory function of the carotid bodies has been proposed to explain tachypnea in this case (Fernandez et al. 2008).

### Short‐term food deprivation diverts metabolism away from glycolysis and attenuates the RER rise in endotoxic shock

**Figure 7:**
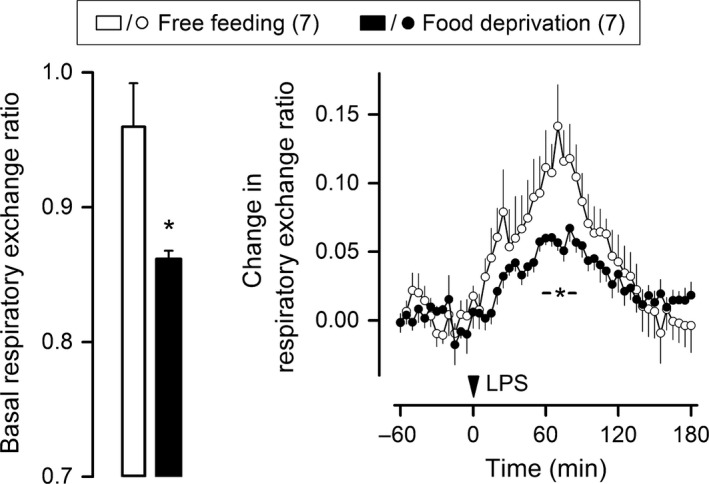
Short‐term food deprivation attenuates the acidosis‐associated rise in respiratory exchange ratio. This experiment was conducted at an ambient temperature of 22°C. Lipopolysaccharide (LPS) O127:B8 (0.5 mg/kg) was administered via IV catheter extensions to rats that had been subjected to 24 h of food deprivation or to rats with unrestricted access to food. The number of animals in each group is shown in parentheses. The basal level of the respiratory exchange ratio (left panel) was estimated over the 30‐min period that preceded an injection. The response to LPS (right panel) is shown as change from the baseline level. Data are means ± standard errors. The time of the LPS injection is marked by an arrowhead. An asterisk (with or without a horizontal bar) denotes a statistical difference (*P *<* *0.05) between the groups.

This experiment was conducted at a T_a_ of 22°C. A 24‐h food deprivation regimen was employed to divert metabolism away from glucose oxidation and toward fatty acid oxidation. The effectiveness of this strategy was confirmed by the fact that the basal RER of the food‐deprived rats was intermediate between the respiratory quotients for fatty acid oxidation (0.7) and glucose oxidation (1.0), whereas the basal RER of the free‐feeding rats was much closer to the respiratory quotient for glucose oxidation (Fig. 7). This switch in metabolic fuel utilization was associated with a significant attenuation in the RER rise induced by LPS O127:B8 (0.5 mg/kg). The amplitude of the response was the most affected parameter: on average, the RER of the food‐deprived rats was increased by only 0.06 at 70 min post LPS, an attenuation of 58% compared to free‐feeding rats.

**Figure 8:**
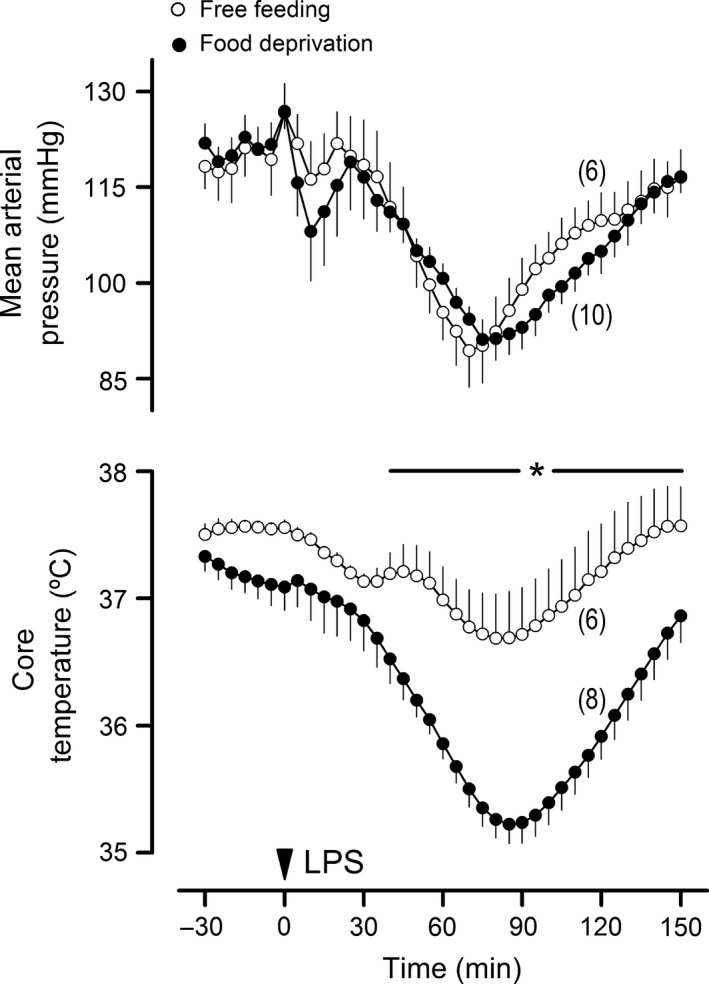
Lipopolysaccharide (LPS)‐induced hypotension and hypothermia were not attenuated by the same food deprivation regimen that lessened the acidosis‐associated rise in respiratory exchange ratio. LPS O127:B8 (0.5 mg/kg) was administered via IV catheter extensions to rats that had been subjected to 24 h of food deprivation or to rats with unrestricted access to food. The number of animals in each group is shown in parentheses. Data are means ± standard errors. The time of the LPS injection is marked by an arrowhead. The asterisk‐containing bar denotes the time window in which there was a statistical difference (*P *<* *0.05) between the groups.

To rule out the possibility that attenuation of acidosis in the food‐deprived animals could have reflected a generalized attenuation of the systemic inflammatory reaction, two other key components of the response to LPS were assessed in this experiment: hypotension and hypothermia. As in previous studies (Yilmaz et al. 2008a,b), LPS‐induced hypotension consisted of two phases: the first phase began only a few minutes after LPS injection, and had a low magnitude and a short duration; the second phase began immediately after the first phase, and was much more pronounced and longer lasting (Fig. 8). None of these phases were affected in the food‐deprived rats, as compared to the free‐feeding controls. Regarding LPS‐induced hypothermia, the data clearly show that this response was not attenuated in the food‐deprived rats; on the contrary, it was exaggerated (Fig. 8). Linear correlation analysis further showed that the magnitude of hypothermia was not a predictor for the attenuation of the LPS‐induced RER rise in the food‐deprived rats (*r* ^2^ of 0.28).

## Discussion

### RER as a novel aid to track H^+^ production in endotoxic shock

To date, the course of acidosis in endotoxic shock has been investigated by serial blood draws. As much as this method is accurate, it provides data with poor temporal resolution. Additionally, the need for serial blood draws has favored experimentation under anesthesia in all species investigated: dog (Jacobs et al. 1982; Evans et al. 1984), sheep (Dubin et al. 2005), pig (Andersson et al. 2006), cat (Parratt and Sturgess 1974), rat (Schaefer et al. 1995; Scheiermann et al. 2011), and mouse (Sand et al. 2015). These studies led to the notion that acidosis is a progressive phenomenon in endotoxic shock, but this notion must still pass scrutiny in studies with better temporal resolution. It is also possible that anesthetics interfere with the course of LPS‐induced acidosis, given their known influence on inflammation (Spentzas et al. 2011; Gokcinar et al. 2013; Tanaka et al. 2013), metabolic function (Brunner et al. 1975; Ohlson et al. 2004), and acid–base regulation (Arfors et al. 1971; Bonhomme et al. 2009). Another aspect to consider is that anesthesia is often used in conjunction with mechanical ventilation, which inevitably overrides the respiratory component of acid–base regulation. Indeed, Simons et al. (1991) have reported an early respiratory alkalosis in unanesthetized rats infused with LPS, but it is unknown if the dose infused in that study was sufficiently high to promote severe systemic inflammation and shock.

This study introduces RER as a correlate of H^+^ production during the early phase of endotoxic shock in unanesthetized rats. Acute rises in RER were observed consistently following LPS administration, regardless of the LPS serotype or the shock‐inducing dose used. During a response, RER often exceeded the upper limit of the respiratory quotient for metabolic fuel oxidation – which is 1.00 (Peronnet and Massicotte 1991) – thus indicating the genesis of a nonmetabolic load of CO_2_. RER rises of this magnitude have been reported during exercise, and in that case, the nonmetabolic load of CO_2_ is known to arise from an H^+^‐induced equilibrium shift in the bicarbonate buffer (Naimark et al. 1964; Beaver et al. 1986). To validate the applicability of this model to endotoxic shock, our first step was to confirm two of its chief corollaries: (1) that decreases in pH mirror the rise in RER; and (2) that [HCO_3_^−^]_a_ is depleted proportionally to the degree of acidosis. Not only did the data confirm these corollaries, but they also showed that RER was quite sensitive to reveal mild acid–base disturbances. One might argue that the fall in [HCO_3_^−^]_a_ could have resulted from the renal loss of this anion, but this alternative scenario is not consistent with the genesis of nonmetabolic CO_2_ and rise in RER. The second step in the model validation was to rule out the possibility that a respiratory acidosis could be taking place. Accordingly, we demonstrated that P_a_CO_2_ was never increased during the course of the experiments; P_a_CO_2_ was even reduced as a consequence of hypometabolism when LPS was given to rats at the T_a_ of 22°C. The third step was to rule out the contribution of V_A_ adjustments to the observed changes in RER. By impacting alveolar CO_2_ exchange more than O_2_ exchange, elevations in V_A_ can raise RER (Chin et al. 2007). This mechanism, however, is unlikely to have played a role in the RER response to LPS for two reasons: (1) hyperventilation‐related increases in RER are associated with alkalosis and not with acidosis; and (2) T_a_‐dependent changes in the V_A_ response to LPS had no impact in the RER and pH responses. The fourth, and last, step in the model validation consisted of obtaining clues about kidney function. In this respect, our data indicate that LPS may be inducing K^+^ loss. Taken together with the fact that K^+^ excretion is usually coupled with H^+^ excretion in hypodynamic states like endotoxic shock (Jacobson and Seldin 1983), these data can be viewed as an indirect sign that the kidneys are losing more H^+^ in an effort to compensate for the developing acidosis. Therefore, the results of this four‐step model validation are consistent with a nonrespiratory, nonrenal origin for acidosis in early endotoxic shock.

Since RER can be measured noninvasively at a high sampling rate, its use as a correlate of acidosis in endotoxic shock may yield new insights into the dynamics of the response. Indeed, this study revealed phasic rises in RER following administration of LPS to unanesthetized rats. Such rises started only 5–20 min post LPS, peaked at 30–70 min, and ended at 120–180 min. The response pattern was monophasic with LPS doses of 0.5 and 1 mg/kg, but a biphasic pattern was evident with the dose of 2 mg/kg. The associated fall in pH was similarly phasic and consistent with the timing of the RER response. These observations contrast with those reported for anesthetized animals, in which acidosis was portrayed as a progressive phenomenon (Parratt and Sturgess 1974; Jacobs et al. 1982; Evans et al. 1984; Schaefer et al. 1995; Dubin et al. 2005; Andersson et al. 2006; Scheiermann et al. 2011; Sand et al. 2015). Interestingly, there is evidence to suggest that other responses to LPS have distinct time‐courses in anesthetized versus unanesthetized animals. For example, whereas LPS‐induced hypotension is usually progressive in anesthetized rats (Leach et al. 1998; Macarthur et al. 2000; Miura et al. 2000; Vayssettes‐Courchay et al. 2002), there are plenty of reports of it having a phasic pattern in unanesthetized rats (Chang et al. 1987; Romanovsky et al. 1996; Knuepfer et al. 2004; Steiner et al. 2009; Al‐Saffar et al. 2013). As another example, the regulated hypothermia that naturally replaces fever in the early stage of endotoxic shock is often transient in unanesthetized rats (Romanovsky et al. 1996; Almeida et al. 2006a; Corrigan et al. 2014), as appears to be the case for the hypothermia that develops in humans with severe sepsis (Fonseca et al. 2016). This thermometabolic response is usually absent in experiments performed under anesthesia, owing to the disruption of thermoregulation by anesthetics and to the use of external heating to clamp T_c_ at ~37°C.

### Mechanisms of metabolic acidosis in endotoxic shock

A nonrespiratory, nonrenal origin for acidosis in early endotoxic shock agrees with studies indicating a heightened metabolic production of H^+^ (Fink et al. 1989; VanderMeer et al. 1995; Oldner et al. 1999). In this scenario, it was reasonable for us to postulate that there could be interdependence between thermometabolic adjustments and acidosis in endotoxic shock (see Introduction). However, to our surprise, the acidosis induced by LPS remained unchanged regardless of whether $\dot{V}$O_2_ and T_c_ fell at a T_a_ of 22°C or did not fall at a T_a_ of 29°C*. One possible explanation for the dissociation between $\dot{V}$O_2_ and acidosis is that thermometabolic responses and H^+^ production depend on distinct tissues: whereas brown fat is thought to play a central role in thermometabolic adjustments (Romanovsky et al. 2005), the gut has been shown to be a source of H^+^ in endotoxic shock (Fink et al. 1989; VanderMeer et al. 1995; Oldner et al. 1999). But, it should be noted that this explanation does not account for the dissociation between T_c_ and acidosis, given that a change in T_c_ should affect local temperatures in the gut as well as in other organs.

These observations also have implications to the relationship of acidosis with tissue hypoxia. This is because Corrigan et al. (2014) have provided evidence indicating that the early downregulation of $\dot{V}$O_2_ and T_c_ in response to LPS serves as a preemptive strategy to maintain the balance between oxygen delivery and demand, thus obviating tissue hypoxia. Such downregulation is manifested in a cool environment (subneutral T_a_), which is also preferred by rats during the early phase of endotoxic shock (Romanovsky et al. 1996; Almeida et al. 2006a,b). By the same token, forced exposure to a warm environment (neutral or supraneutral T_a_) prevents $\dot{V}$O_2_ and T_c_ from falling, and in that case, tissue hypoxia ensues (Corrigan et al. 2014). Hence, the fact that LPS‐induced acidosis was not modified by T_a_‐dependent thermometabolic adjustments in this study argues in favor of its independence of tissue hypoxia. This new line of evidence adds to studies in which volume expansion‐driven increases in oxygen delivery failed to attenuate acidosis in endotoxic shock (Schaefer et al. 1995; VanderMeer et al. 1995; Oldner et al. 1999; Aksu et al. 2012).

Another important finding of this study is that short‐term food deprivation attenuates the acidosis‐related rise in RER. This effect did not result from a general attenuation of the inflammatory reaction, since food deprivation did not attenuate two other pivotal components of the systemic inflammatory response to LPS, namely hypotension and hypothermia. The hypothermic component was even exaggerated, an observation that is in line with a previous study (Krall et al. 2010). In that study, 24 h of food deprivation exerted no influence on the levels of proinflammatory cytokines in early endotoxic shock, and the enhancement of hypothermia was proposed to be the result from a greater responsiveness to inflammatory mediators that lower T_c_, such as prostaglandin D_2_. In the face of these facts, diversion of metabolism away from glycolysis in the food‐deprived rats poses as the most plausible explanation for their attenuated H^+^ production in response to LPS.

To date, this is the most direct evidence for the involvement of glycolysis in LPS‐induced H^+^ production. Previous studies on the subject were based on indirect evidence and on the notion that lactic acid produced via anaerobic glycolysis is the main source of H^+^ under this circumstance. Although this notion has become quite popular, it is nonfactual considering the evidence contrary to the involvement of hypoxia in LPS‐induced acidosis (as discussed above), and the evidence indicating that lactate is not a source of metabolic H^+^ (Robergs et al. 2004). On the other hand, the steps of glycolysis upstream of lactate production are known to be a significant source of H^+^ (Robergs et al. 2004). Hence, aerobic glycolysis can be the main source of H^+^ during endotoxic shock. In agreement, aerobic glycolysis has been shown to be activated in skeletal muscle during experimental endotoxic shock (James et al. 1996) and clinical sepsis (Gore et al. 1996; Levy et al. 2005), possibly as the result of an increased sympathetic outflow (Levy et al. 2008). We still have much to learn about sympathetic function in endotoxemia and sepsis, but it is becoming evident that it can be changed in a diverse, organ‐specific fashion (Rogausch et al. 1997; Ramchandra et al. 2009). Hence, it is not inconceivable to think that an increased sympathetic outflow to muscle and gut could contribute to H^+^ production, while a decreased sympathetic outflow to brown fat could lower whole‐body $\dot{V}$O_2_ and T_c_. This is, of course, speculative at this moment.

### Concluding remarks and perspectives

This study identifies RER as an aid to noninvasively monitor the course of metabolic H^+^ production during the early phase of endotoxic shock in rats. This new aid revealed that, in the absence of anesthesia, acidosis is a phasic rather than a progressive phenomenon in this model. We then examined whether H^+^ production could be caused or countered by the downregulation of $\dot{V}$O_2_ and T_c_ that protects tissues from hypoxia in unanesthetized rats. This possibility was rejected by the fact that acidosis remained unaffected when the hypometabolic, hypothermic response was allowed or prevented by exposure to different thermal environments. Last, but not least, this study shows that H^+^ production in endotoxic shock is more pronounced when glucose is the predominant substrate that fuels aerobic metabolism. Collectively, these findings indicate that excess H^+^ production in unanesthetized rats with endotoxic shock results from a phasic activation of glycolysis, which occurs independently of physiological changes in mitochondrial oxidation and T_c_.

Future studies will be warranted to evaluate whether RER serves as a tool to track H^+^ production in other models of systemic inflammation or sepsis. There is evidence that acidosis in a polymicrobial model of sepsis can occur as early as in endotoxic shock, presumably preceding respiratory and renal derangements (Scheiermann et al. 2011). This earliest phase of the response is a window of opportunity for studies aimed at uncovering the fundamental mechanisms of metabolic H^+^ production in systemic inflammation. In our view, such studies hold the greatest potential for readily using RER to track H^+^ production. This point of view does not imply that RER bears no value to probe for H^+^ production in the later stages of systemic inflammation as well as in septic patients, but it should be considered that some hurdles will need to be overcome before RER can be used for this purpose. More specifically, acidosis in late systemic inflammation is often multifactorial, and some of the factors involved can mask, at least partly, the impact of H^+^ buffering on RER. This can happen, for example, when lung injury impairs gas exchange. Tools to overcome this and other hurdles include more accurate and sensitive respirometers as well as statistical methods to distinguish rapid H^+^‐related changes in RER from slower trends related to other factors.

## Conflict of Interest

No conflicts of interest, financial, or otherwise, are declared by the authors.

## Note

1 For more information on the T_a_‐dependence of the thermometabolic responses to LPS, see Corrigan et al. (2014); Krall et al. (2010); Liu et al. (2012); Steiner et al. (2004); Steiner et al. (2009).
